# Biomedical Radioactive Glasses for Brachytherapy

**DOI:** 10.3390/ma14051131

**Published:** 2021-02-27

**Authors:** Francesco Baino, Elisa Fiume, Sara Ciavattini, Saeid Kargozar, Roger Borges, Luis A. Genova, Juliana Marchi, Enrica Verné

**Affiliations:** 1 Institute of Materials Physics and Engineering, Department of Applied Science and Technology (DISAT), Politecnico di Torino, 10129 Torino, Italy; elisa.fiume@polito.it (E.F.); sara.ciavattini@studenti.polito.it (S.C.); enrica.verne@polito.it (E.V.); 2 Interdepartmental Center PoliTO BIOMedLab, Politecnico di Torino, 10129 Turin, Italy; 3 Interdepartmental Center J Tech@PoliTO, Politecnico di Torino, 10129 Turin, Italy; 4 Department of Mechanical and Aerospace Engineering (DIMEAS), Politecnico di Torino, 10129 Torino, Italy; 5 Tissue Engineering Research Group (TERG), Department of Anatomy and Cell Biology, School of Medicine, Mashhad University of Medical Sciences, Mashhad 917794-8564, Iran; kargozarsaeid@gmail.com; 6 Centro de Ciências Naturais e Humanas, Universidade Federal do ABC, 09210-580 Santo André, SP, Brazil; roger.borges@aluno.ufabc.edu.br (R.B.); juliana.marchi@ufabc.edu.br (J.M.); 7 Centro de Ciência e Tecnologia dos Materiais, Instituto de Pesquisas Energéticas e Nucleares, 05508-000 Sao Paulo, SP, Brazil; lgenova@ipen.br

**Keywords:** durable glasses, bioactive glasses, radioactive, radioisotope, microspheres, cancer treatment

## Abstract

The fight against cancer is an old challenge for mankind. Apart from surgery and chemotherapy, which are the most common treatments, use of radiation represents a promising, less invasive strategy that can be performed both from the outside or inside the body. The latter approach, also known as brachytherapy, relies on the use of implantable beta-emitting seeds or microspheres for killing cancer cells. A set of radioactive glasses have been developed for this purpose but their clinical use is still mainly limited to liver cancer. This review paper provides a picture of the biomedical glasses developed and experimented for brachytherapy so far, focusing the discussion on the production methods and current limitations of the available options to their diffusion in clinical practice. Highly-durable neutron-activatable glasses in the yttria-alumina-silica oxide system are typically preferred in order to avoid the potentially-dangerous release of radioisotopes, while the compositional design of degradable glass systems suitable for use in radiotherapy still remains a challenge and would deserve further investigation in the near future.

## 1. Radiation Therapy: A Short Overview

Radiation therapy involves the treatment of solid tumors by using ionizing radiation as a physical therapeutic agent in order to destroy tumoral cells, thus reducing the size of the malignant mass [1]. Specifically, radiation therapy aims at damaging the DNA of cancer cells so that they lose the capability to divide and proliferate, thus leading to the cell death process [2]. Radiation forms ions and stores energy in the cells of the tissues that are exposed to the ionizing beam [3], which can have a direct or indirect effect (damage) on DNA. The latter damage is associated to the production of reactive oxygen species and comes from the ionization or excitation of the water molecules in the cells [2].

Four major types of cell death induced by radiation have been described in the literature, i.e., (i) apoptosis, (ii) mitotic cell death, (iii) necrosis, and (iv) autophagy [2,4,5,6].

Besides causing cell death, radiation can also lead to cell senescence, which is the permanent loss of proliferative capacity [6]. Thus, senescent cells are still alive but are unable to divide, synthesize DNA, spread, and flatten [2]. Radiation therapy does not kill cancer cells immediately, but several hours, days, or even weeks may be needed before tumor cells start to die depending on pathology and treatment conditions, mainly related to dosage adjustment. As a result, tumor cells continue to die for weeks to months after radiation therapy ends [2].

Radiation therapy can cause damage to both normal and cancer cells, but healthy cells are able to self-repair faster than neoplastic ones and to maintain their function [7].

**Figure 1:**
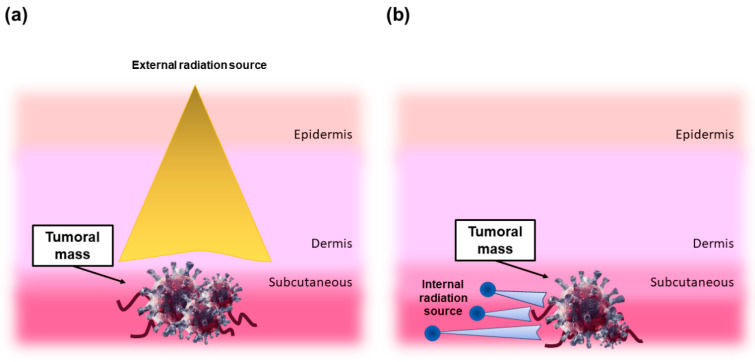
Principles of radiation therapy. (**a**) Scheme demonstrating tissue absorption of external beam radiation: the radiation emitted damages both tumor and healthy cells through electron excitation and release of energy. (**b**) The concept behind brachytherapy: radioactive seeds (blue spheres) emit beta and/or gamma rays into the target tumor.

Radiation can be delivered to the injured site by *ab externo* or *ab interno* strategies. In the first case, external beam radiation is delivered from outside the body to the location of the tumor. This is the most commonly-used route in the clinical practice and typically employs high-energy gamma rays (cobalt unit), or, in more advanced and efficient applications, a linear accelerator that can provide high-energy X-rays or electrons. (Figure 1a) [2]. In the second case, internal radiation is delivered from inside the body directly at the site of the cancerous mass by means of a radioactive source, which can be either placed directly at the tumor site or targeted by specific physical-chemical mechanisms. As an example, in unsealed source radiotherapy (or unsealed source radionuclide therapy (RNT)), radioactive chemical substances (called radiopharmaceuticals) are administered by injection or ingestion with minimal invasive strategies and targeted to the tumor site by, for instance, antigen/antibody interactions.

As regards to brachytherapy, the radioactive sources (or radioisotopes) are sealed/immobilized in seeds, pellets, wires, or microspheres capsules (which can be eventually removed after the treatment) that beneficially prevent the radioisotope from moving or dissolving in body fluids (Figure 1b) [2].

There is a wide range of radionuclides that can be used in brachytherapy. Some important physical aspects, such as the emitted radiation, their associated average energy, the half-life, and the emitted dose rate, in addition to the patient’s clinical conditions and disease stage, must be considered to choose the best option. In general, the radioisotope must have a radiation spectrum that allows the treatment of tumors with different dimensions. Thus, an ideal radioisotope should be beta-ray emitter, with high energy, high activity, and short half-life, able to penetrate deeply up to 12 mm in living tissue. Moreover, this radioisotope should also emit low-energy gamma radiation, thus identifying the site of the radioactive source inside the body (through gammagraphy), and monitoring its biodistribution. Several beta-emitting radioisotopes have suitable characteristics to be applied in brachytherapy for the treatment of different tumors. Some studies regarding the use of alpha radiation for cancer treatment have also been reported in the recent literature [8,9,10].

Radioactive sources can be left in situ indefinitely—which is known as permanent brachytherapy—or be removed and periodically replaced in order to preserve their therapeutic action and/or minimize side effects, according to a process called temporary brachytherapy [11].

In situ irradiation is a powerful technique and includes many advantages, such as the possibility to use a shorter-range radiation (typically a beta-ray emitter) that minimizes the damage to adjacent, healthy tissue as compared to employing an external source. Therefore, larger doses of radiation can be safely and locally delivered, which remarkably increases the success of destroying the tumor [12].

This approach is commonly used in the treatment of soft tissue cancers, like gynecological and prostate malignant tumors, as well as in situations where recurrent treatments are indicated [2]. In the field of osseous tumors, radiation therapy has been successfully used for the management of Ewing’s sarcoma [13] and bone metastatic cancer [14]. In this regard, Feng et al. [14] reported that implantable seeds containing Iodine-125 (^125^I) as a radioactive element are useful for the palliative treatment of osseous metastases by brachytherapy. After implantation of ^125^I seeds, patients showed significant reduction in cancer-associated pain and improvement in quality of life [14].

**Figure 2:**
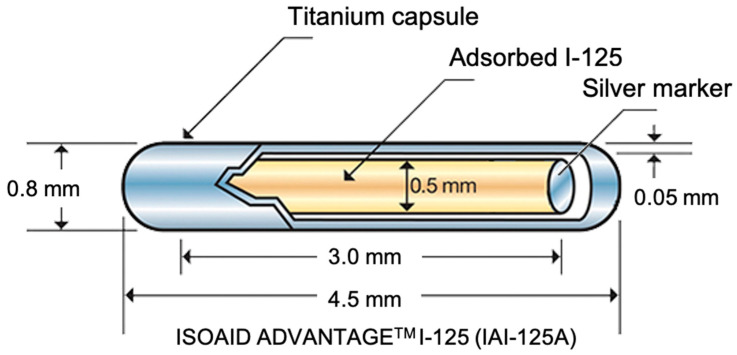
Example of a commercial, clinically-used seed for brachytherapy [17].

Most commercialized seeds for clinical brachytherapy are composed of a titanium capsule [15] which is usually less than 5 mm in length and 0.8 mm in diameter (Figure 2) [16].

Titanium is a suitable material for this purpose since it is non-toxic, non-degradable, and shows good biocompatibility [15]. This capsule contains the radioactive source and is used to avoid direct contact between radioactive material and patient’s body fluids and tissues [16]. The capsule also contains a long silver rod that is used as a radiographic marker for correct positioning and localization [18]. The isotope ^125^I has a half-life of about 59.5 days and decays by electronic capture with X-rays or gamma-rays emission. The electrons emitted during the decaying are absorbed by the titanium capsule [18].

Irradiation can be used in combination with other therapies such as surgery, chemotherapy, or immunotherapy [2]. Radiation therapy administered before surgery is called neoadjuvant therapy and aims at reducing the tumor size. On the contrary, if administered after surgery, radiotherapy is called adjuvant therapy and aims at destroying the residual cancer cells [2].

The most common side effects of radiation therapy include patient’s fatigue, alopecia, skin burns, nausea, diarrhea, and risk of secondary cancers due to ionization in healthy cells and tissues [19].

## 2. Why Using Radioactive Glasses in Brachytherapy?

As already discussed in the Section 1, brachytherapy is a kind of radiotherapy for solid cancer treatment that, in general, consists in placing the radiation source inside the tumor area in order to deliver a therapeutic dose of beta-radiation emission into the cancerous tissue in situ, thus limiting the damage to the surrounding healthy tissues [20]. Most commercial seeds for such an application are composed of a metallic capsule containing ^125^I as a radioactive element [21]. Titanium, which tends to self-passivate with an ultra-hard and chemically-inert thin film of TiO_2_ in the biological environment, is an excellent option for making the capsule. This approach, however, has a couple of limitations: (i) a second invasive surgery may be required for the removal of the capsule and (ii) ^125^I has a long half-life (59.5 days) [22].

Yttrium-90 (^90^Y) is an attractive alternative radioisotope for this purpose since it can be obtained by neutron activation of ^89^Y, which is abundant in nature, has a shorter half-life of 64.2 h as compared to ^125^I, and its emitted beta radiation has an average range in soft tissues of only 2.5 mm, thereby minimizing the radiation that reaches healthy tissues [23,24]. Besides ^90^Y, other beta-emitting radionuclides are used for cancer treatment, as summarized in Table 1.

Radioactive microspheres can be delivered to a target organ either through the bloodstream or by direct injection in the tumor area [23]. In the 1960s, polymeric microspheres coated with radioactive ^90^Y were first used to treat liver cancers in situ after being injected into the hepatic artery. However, the study was suspended soon because the radionuclide ^90^Y could not be confined [23].

In order to overcome this issue and successfully increase the life expectancy of the patients, new materials have been studied over the following years [24,25]. In this regard, special glass compositions have emerged as attractive materials for brachytherapy, relying on the concept that radionuclides can be safely incorporated in a biomedical glass matrix [12]. Currently, there are two commercial products containing ^90^Y as the incorporated radionuclide, both available as microspheres: SIR-Spheres^®^, a polymeric product marketed by Sirtex Medical, and TheraSphere^®^, a glass product developed by MDS Nordion.

There are two procedures to prepare a radioactive glass for in vivo use. The first method consists in mixing the radioactive agent with the batch material and then process the blend; in this way, the radioisotope becomes an integral part of the glass. The main disadvantage is related to the safety measures that must be taken to manage radioactive materials during the processing, such as melting and quenching [12]. The second approach, which is the most commonly-used, involves the production of a conventional glass by using non-radioactive raw materials followed, as the last step in the fabrication process, by neutron activation, thus making the glass radioactive. This method simplifies the processing of the glass but limits the chemical composition of the batch: specifically, oxide glasses containing Na_2_O, K_2_O, and CaO—which are common glass network modifiers—should be avoided as some neutron-activated radioisotopes of Na, K, and Ca have dramatically long half-life (thousands of years), making these materials unsuitable for medical applications [12].

**Figure 3:**
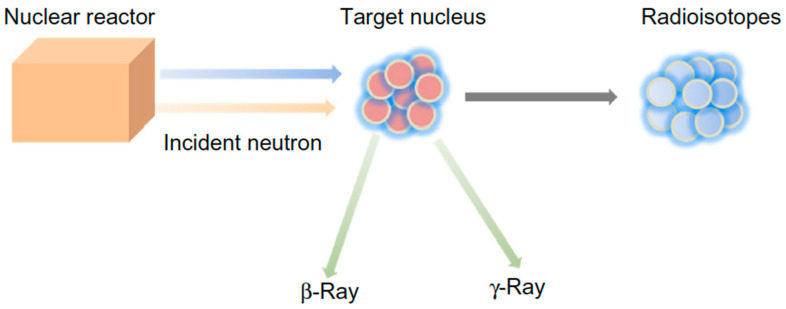
Schematic representation of nuclear reaction between a target nucleus and an incident neutron, leading to the formation of a radioisotope and the emission of beta and/or gamma rays. Figure reproduced from Deliberato Aspasio et al. [27].

Despite the great potential exhibited by glasses in radiotherapy, research on this topic is still relatively uncommon and new materials are in the early stage of development. This is probably due to the fact that few research groups around the world have nuclear facilities to produce biomedical glasses containing radioisotopes, focusing the study on radioactive properties. In fact, the main experimental procedure to impart radioactive properties to glasses is neutron activation, whose principle of operation is shown in Figure 3. This strategy requires the availability of a nuclear reactor to generate a neutron beam to which the glass containing the desired isotope will be exposed; hence, radioisotopes and beta and/or gamma rays are obtained as reaction products [26].

In general, the concentration of the neutron-activatable element in the glass should be high enough to elicit the level of specific therapeutic activity recommended for the chosen treatment, and such effect may depend on the element being activated, the organ being treated, the neutron flux, and activation time [23].

In order to be used for tumor brachytherapy, biomedical glasses should satisfy three major requirements, namely (i) biocompatibility [23], (ii) chemical durability of the glass matrix and resistance to body fluids for avoiding radioisotope release in the patient’s body [23], and (iii) absence of other elements able to form undesired radioisotopes with long half-life during the neutron activation process [23]. Referring to point (ii), it is worth mentioning that the physiological pH value of human body fluid is about 7.4, but it can have lower values in the presence of tumors; hence, the glass should have high chemical durability even under acidic conditions and should not release active radioisotopes into normal tissue.

Over the years, however, the need for high durability of the glass has become less stringent and some researchers also proposed the use of partially-soluble glasses for brachytherapy, thus combining anticancer properties (via in situ irradiation) and bioactivity (via ion dissolution).

## 3. Non-Degradable Glasses

### 3.1. Compositions and Properties

The need for using neutron activation and having a glass with high chemical durability has initially rejected most of the commonly-used bioactive glasses because they form undesirable radioisotopes (e.g., Na and Ca) during neutron activation and are prone to fast dissolution in aqueous media.

Thus, rare earth aluminosilicate (REAS) durable glasses with simple composition have been considered potentially suitable for this application. They are composed of only three oxides: alumina (Al_2_O_3_), silica (SiO_2_), and the neutron activatable rare earth oxide (RE_2_O_3_). During neutron activation, radioisotopes from aluminum (Al), silicon (Si), and oxygen (O) are also formed but they decay very quickly without any adverse effect for the human body [12].

**Figure 4:**
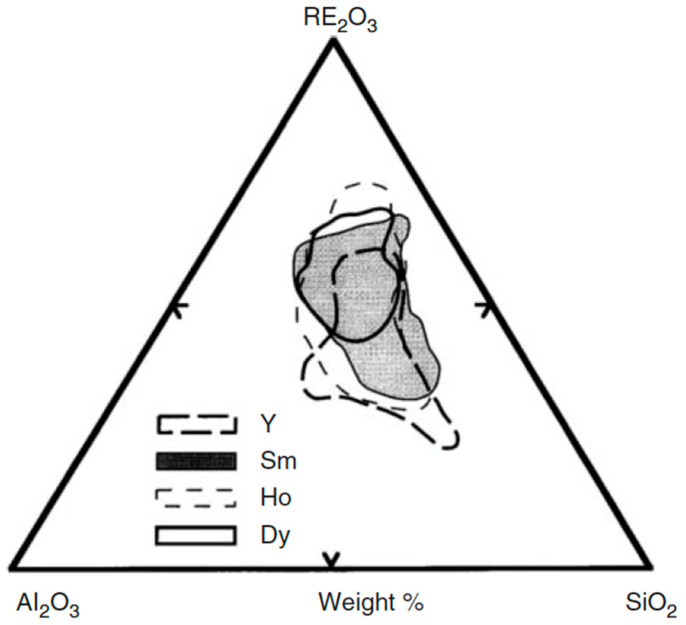
Compositional ternary diagram showing the glass formation range for REAS compositions that melt below 1600 °C. Yttrium, while not being rigorously a rare earth element, is comprised in the diagram because it has similar properties to those of rare earths [12].

Compositional regions that allow the formation of the glass for various families of REAS glasses having melting temperature below 1600 °C are shown in Figure 4 [12].

REAS glasses for radiotherapy typically contain rare earth oxide in the range of 32 to 69 wt.%. The density of REAS glasses increases with increasing RE_2_O_3_ concentration. These materials are characterized by an exceptional chemical durability in the biological environment, which generally does not allow the loss or release of any radioisotope [12].

**Figure 5:**
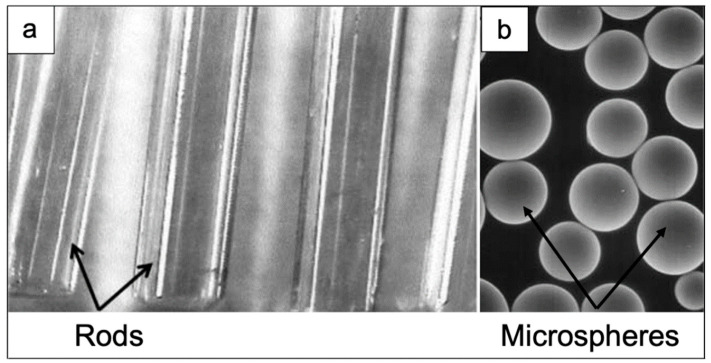
REAS glasses with different shapes: (**a**) rods and (**b**) microspheres, used in brachytherapy. The rods in (**a**) and the glass microspheres in (**b**) were based on the 46.8Sm_2_O_3_-18.2Al_2_O_3_-35SiO_2_ (wt.%) and 55Y_2_O_3_-20Al_2_O_3_-25SiO_2_ (wt.%) systems, respectively. Rods in figure (**a**) have a diameter of 1 mm, while the spheres depicted in figure (**b**) exhibit a diameter ranging between 20–30 μm [12].

REAS glasses used as radiation delivery vehicles are commonly produced in the form of microspheres with diameter ranging from 10 to 30 μm, although larger glass seeds or fibers can be used as well (Figure 5).

The use of microspheres having no sharp edges is preferred from an operative viewpoint due to the relative ease of both intravenous administration and direct injection into the target site. The surface of glass microspheres is smooth in order to prevent any damage to the delicate walls of blood vessels and their size can be controlled and properly tailored for a particular organ, thus allowing a certain degree of customization. In this way, the microspheres can flow through the larger vessels without passing the capillary bed of the target organ, thus being accumulated in the tumor [12]. As an example, considering liver cancer, microspheres are administered directly to liver tumor via hepatic artery. Microspheres in the range of 20 to 30 μm are small enough to go through hepatic arteries, but too large to pass through smaller blood vessels within the tumor, where they remain permanently positioned, thus compromising the vascularization of the tumoral tissue while emitting therapeutic radiation.

REAS microspheres incorporating beta-emitting ^90^Y, ^153^Sm, ^165^Dy, ^166^Ho, and ^186^Re/^188^Re were tested in animal models. It was shown that in situ beta radiation from REAS glasses carrying ^166^Ho or ^90^Y exhibited a dual function in both stopping the tumor growth and reducing the size of tumor mass. The effectiveness of in situ radiation depends first of all on the tumor features (type, location, dimension, stage, and vascularization), the range and energy of the radiation, the half-life and amount of the radioisotope, and thus, it has to be customized [12].

To date, only REAS glasses containing a single type of radioisotope have been produced and experimented for the in situ irradiation of a target organ. However, combining different neutron-activatable radioisotopes into a single glass microsphere could lead to some advantages regarding the optimization of the irradiation of tumors of different sizes and the delivery of the radiation dose over a prolonged period of time. Another option could be the mixing of two or more REAS glasses, each one containing a distinct neutron-activatable element. As a result, the delivered dose will be a combination of radiation types, energies, and half-lives [12].

The most famous example of REAS glasses are yttria-alumina-silica (YAS) glasses with up to 55 wt.% Y_2_O_3_, which were proved to have an excellent chemical durability and can be produced in the form of microspheres (25–35 μm) by flame spheroidization method [23,24,28]. The starting non-radioactive YAS glass contains the ^89^Y isotope and is then activated by neutron bombardment. In 1988, Day et al. [29] patented the application of ^90^Y-containing biocompatible glass microspheres for radiotherapy, which enabled the development of a new treatment for liver cancer, combining radiation and embolization effects of the capillaries [29]. The ^90^Y-containing microspheres safely deliver a large dose of radiation (up to 15,000 rad) to the tumor and approximately 2 to 8 million glass microspheres deposit in the capillary bed after being injected into the hepatic artery, thus also reducing the blood flow to the malignant tumor by a radioembolization effect [23,28]. The comparison with external radiotherapy in terms of dose administered—and hence therapeutic efficacy—is impressive, as a maximum of 3000 rad can be tolerated by patients undergoing conventional external irradiation [2]. As ^90^Y is a short-range beta-emitter (2.5–3 mm in the liver), irradiation is confined locally at the tumor site. Moreover, animal studies have shown that YAS glass in the 40Y_2_O_3_-20Al_2_O_3_-40SiO_2_ (wt.%) system did not release any detectable amount of ^90^Y [28]. The first clinical trial reported by Boos et al. [30] documented a significantly positive response in 35 out of 46 patients suffering from hepatocarcinoma, with complete remission of 1, partial remission of 6, and stability of the disease of 24. Moreover, the mean survival was 16.1 months for the responsive versus 8.8 months for unresponsive patient. After being approved by the Food and Drug Administration (FDA) in 1999, ^90^Y-containing glass microspheres were marketed under the tradename of TheraSphere^®^ (Boston Scientific Corporation, Watertown, MA, USA) [23]. At present, this product is commercially used in over 200 specialized centers worldwide for the treatment of liver cancer. Once a significant reduction of tumor mass/size has been achieved by the radioembolization process, other follow-up therapies can be performed like surgery or transplants; furthermore, life expectancy in terminal patients has been increased from 5–7 months to 12–24 months [28]. Compared to other cancer treatments like chemotherapy, TheraSphere^®^ yields fewer side effects, just causing flu-like symptoms such as fatigue, a slight fever, or abdominal pain for a few days in a few patients after the treatment [28]. A comprehensive overview of the clinical applications of TheraSphere^®^ for liver cancer therapy was provided by Bretcanu and Evans [31].

Recently, TheraSphere^®^-based treatment has also been proposed to selected patients with metastatic colorectal carcinoma of the liver. The most beneficial effect was found in association with chemotherapy, but even patients with chemotherapy-refractory disease received some benefits from the treatment [32]. In order to corroborate these promising results, a multicenter, randomized, phase 3 trial was launched in 2018 in 100 sites in the USA, Canada, Europe, and Asia for evaluating the efficacy and safety of TheraSphere^®^ radioembolization combined with second-line therapy in patients with metastatic colorectal carcinoma of the liver who had disease progression during or after first-line chemotherapy [33].

### 3.2. Manufacture of Glass Microspheres

Microspheres produced using various materials have been recognized to have a great impact on biomedical progress in a wide range of clinical applications. Table 2 collects a representative—although non-exhaustive—list of the main products based on microsphere technology for biomedical use.

Dealing with glasses and glass-ceramics, silicate, phosphate, and borate microspheres have been successfully produced by several research groups, as comprehensively reviewed by Hossain et al. [49], showing their great potential in fields like drug delivery, bone tissue engineering and regeneration, as well as absorption and desorption of chemical/biological substances. As a result, different manufacturing processes have been developed over the years to produce microspheres with narrow particle size distribution and high accuracy level.

The most widely used methods for obtaining glass microspheres are: (i) flame spheroidization process; (ii) dropping crushed glass down a vertical tube furnace; (iii) sol-gel method.

**Figure 6:**
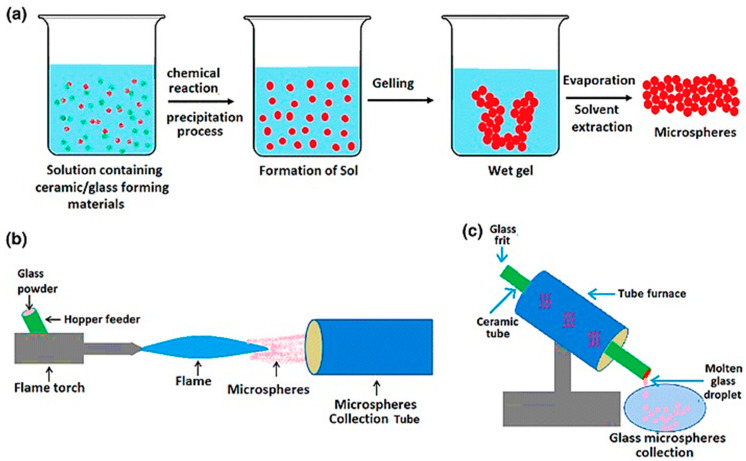
Illustration of glass microsphere production by the (**a**) sol-gel, (**b**) flame spheroidization, and (**c**) furnace methods. Figure retrieved from (Hossain et al. 2015) [49].

A scheme of each of these methods is given in Figure 6 [49].

The flame spheroidization and the vertical tube furnace approach are both based on the same principle. In both methods, irregular glass particles are molten above the melting point and the surface tension upon free fall, only under gravity force, makes the glass droplet to minimize its surface energy by forming spheres [50,51,52]. It is worth noting that both methods depend on the glass melting temperature. The composition design of the microsphere is also considered before producing them to avoid too high melting temperatures that could be difficult to reach in such an experimental apparatus [53,54].

One of the significant differences between the flame spheroidization and the use of a vertical tube furnace is the origin of the heat used to melt the glass, which plays a significant role on other variables that need to be controlled. In a vertical tube furnace, the heat source of the furnace comes from an electrical resistance or coils of magnetic induction [51,53,55]; moreover, the tube length is an essential parameter since the particles need enough time to melt and shape themselves into microspheres [56].

In contrast, in flame spheroidization, heat comes from the flame, which requires the control of more parameters, such as particle size selection before the flame, flame temperature, and time of residence. The particle size selection is usually performed by sieving, selecting a particle size range desired to yield microspheres with appropriate size for brachytherapy. The flame temperature can be controlled by using different gases, such as propane/oxygen, acetylene/oxygen, petrol/oxygen, and natural gas/air flames [52]. The residence time is difficult to control, being a variable that depends on irregular particle weight, which controls the velocity of the particle passing through the flame. Taking all these points together, although the flame spheroidization is a cheaper and relatively easy-to-scale-up technique, the microspheres derived from this method are usually less uniform than those obtained by vertical tube furnace since there are more parameters to be controlled [49].

Regarding the glass microspheres produced by the sol-gel process, the particle shape and size are controlled by methods that require much lower temperature than those needed for melt-derived glass. The sol-gel method is based on alkoxides or acids as precursors of glass formers and nitrates and chlorides as precursors of modifier or intermediate oxides [57]. Regarding REAS glasses, tetraethyl orthosilicate (TEOS) is used as a precursor of SIO_2_, aluminum nitrate as a precursor of Al_2_O_3_, and a rare earth nitrate as a precursor of RE_2_O_3_. The first step of the sol-gel method is the hydrolysis of the alkoxide, which in REAS glasses is the TEOS (Equation (1)) [58]:

(1) $$\equiv Si-O-CH_{3}-CH_{2}\;+\;H_{2}O\to \;\equiv Si-OH\;+\;CH_{3}-CH_{2}-OH.$$

The hydrolysis can be performed in either acid or alkaline medium, although the acidic hydrolysis is more common in the production of microspheres [59]. Other glass precursors like nitrates are also added to the acid medium used in the TEOS hydrolysis. Once TEOS is hydrolyzed, forming silicic acid, the condensation reaction can be performed by changing the pH (acid to alkaline or alkaline to acid, depending on the hydrolysis step), temperature, or aging of the solution. In the condensation step, bridging oxygen bonds are formed between silicon tetrahedrons (Equation (2)), yielding a three-dimensional network that is later grown up to form particles:

(2) $$\equiv Si-OH+\;\equiv Si-OH\;\to \;\equiv Si-O-Si\equiv +\;H_{2}O.$$

When the condensation reaction is triggered (the so-called “sol-gel point”), the solution (sol) jellifies due to the precipitation of amorphous particles that increases the solution viscosity (gel). If no restriction is imposed on the gelation step, the particle formed through condensation reactions keeps growing [60]. However, in order to obtain microspheres, different approaches can be used to limit particle growth, such as emulsification, internal gelation, spray-pyrolysis, and spray drying.

In the emulsification method, hydrophobic liquids are mixed, thus making the sol to form droplets confined in an emulsion; the emulsion is later submitted to thermal treatment for catalyzing the condensation of silicon tetrahedrons or to aging, which is similar to the hydrothermal process. In this method, the microsphere size is controlled by the size of the droplets, which is influenced by the temperature, stirring speed, and organic fraction [61].

**Figure 7:**
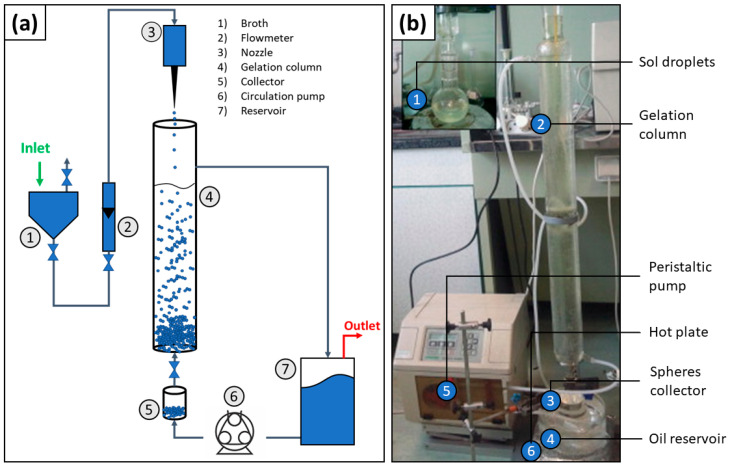
(**a**) Schematic of a generic internal gelation apparatus and (**b**) picture of a typical experimental setup. Figures (**b**) adapted with permission from Poorbaygi et al. [59].

The internal gelation approach is similar to the emulsification method but the sol is dropping into a gelation chamber (Figure 7), which contains hydrophobic organic fluids like ether petroleum or silicone oil [62]. Because the gelation chamber is positioned in a vertical orientation and the sol droplets are formed on the top of the gelation chamber, the droplets fall to the bottom of the chamber; however, because of the oil/water/oil interface, the droplets are confined into spheres to minimize surface energy. Furthermore, while falling, condensation reactions are triggered within the droplets, thereby forming the microsphere. Then, the time spent for a complete condensation reaction depends on how long the droplet keeps falling. This time can be controlled by changing the gelation chamber length, fluid viscosity, and temperature. At the bottom of the chamber, there is a collector of microspheres [59,62].

**Figure 8:**
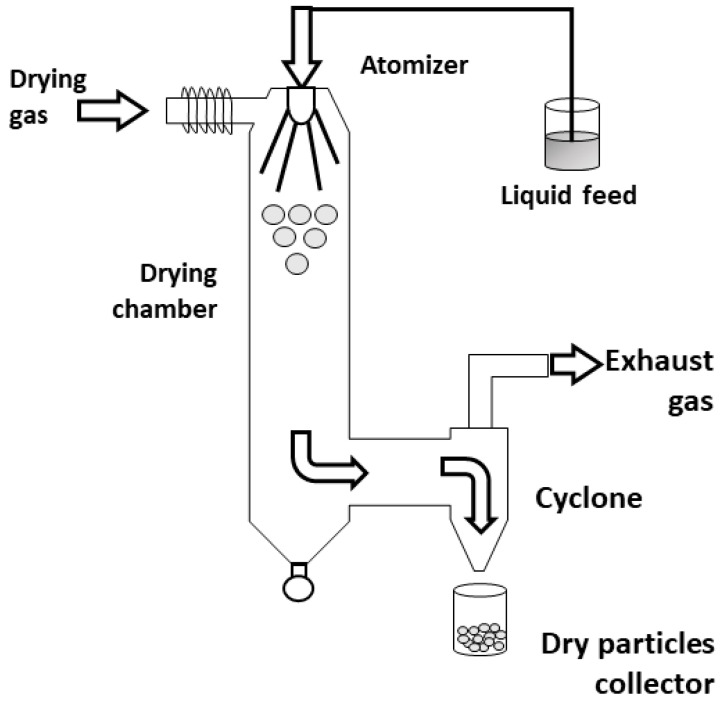
Schematic of spray-drying process. Figure adapted with permission from Sosnik et al. [64].

In the spray-drying method (Figure 8), the sol is atomized in a chamber at a controlled temperature by using inert gases, such as N_2_. Then, the sol droplet is conducted to a cyclone collector, forming an aerosol, where particle selection occurs; in this case, the pressure of the cyclone collector is controlled to allow only aerosols with specific size to “levitate” and pass to the collector, while bigger aerosols are dropped into a product discharge [63].

In all these methods, the dried particles are later submitted to calcination for thermal degradation of residues, incorporation of yttrium and aluminum into the glass structure, and stress relief.

## 4. Biodegradable Glasses

The most peculiar aspect of bioactive glasses is their capability to rapidly dissolve in contact with body fluids, releasing therapeutically active ions into the physiological environment. However, especially dealing with radionuclides, release kinetics have to be properly tuned to be within the safety limits recommended for brachytherapy treatment.

Therefore, caution must always be followed in proposing bioactive glasses for radiotherapy combined to bone regeneration as the need for chemically-stable materials is apparently conflicting with the high reactivity of such glasses. In general, it is highly desired to carefully investigate the influence of radionuclide incorporation on bioactivity and degradation kinetics of the glass in the physiological environment [24]. It is interesting to mention that some special bioactive glasses and glass-ceramics are also used for the treatment of bone cancer via other approaches than the radiation-based ones, such as magnetic induction of hyperthermia [65,66,67]; the interested reader can find more details elsewhere [68].

The first set of biodegradable glasses used as vectors for radioisotopes was developed by Roberto and coworkers in 2003 [26] in order to replace ^125^I seeds used in prostate cancer treatment by brachytherapy. The underlying assumption was that a biodegradable glass would be better for brachytherapy, because titanium-encapsulated ^125^I seeds are temporary and a second surgery is necessary to remove them from the body. The purpose was to develop ternary glasses based on the SiO_2_-CaO system incorporating ^153^Sm radioisotope after neutron activation. ^153^Sm was selected because it has a shorter half-life than ^125^I (46.27 h vs. 54.9 days), thus further proving its suitability for being coupled with a resorbable material with chemical durability lasting a few months. In order to achieve the same activity elicited by ^125^I seeds, it was necessary to incorporate a concentration of samarium between 4.5 and 11.5 wt.% in the glass structure [26]. In 2008, the same research group [69] investigated the degradation of ^153^Sm seeds implanted in rabbit livers by using X-ray radiographic imaging to control the glass durability in vivo. After 7 months, there was no evidence of glass in radiographic images because the seeds had been absorbed in the liver [69].

Cacaina et al. [70,71] demonstrated that bioactive silica-based glasses containing about 5 mol.% of Y_2_O_3_ preserved their bio-reactivity while releasing relatively small amounts of yttrium into simulated body fluid, which is beneficial from a safety viewpoint and supports the suitability of these specific bioactive materials as yttrium vectors in brachytherapy. In addition, it was suggested that yttrium incorporation could even increase the durability of bioactive glasses. Actually, this conclusion was confirmed by analyzing the dissolution rate of bioactive glasses containing significantly different molar fractions of silica and, therefore, the yttrium influence would be overestimated due the heavy dependence of glass durability on silica amount. In fact, bioactive glass compositions with lower amounts of silica were found to comparatively release higher amounts of yttrium and soluble silica into simulated body fluid (SBF) [23].

Apart from silicate glasses, melt-derived alkali-borate and borosilicate glasses deserve to be mentioned in the context of brachytherapy. They degrade progressively in the body within hours or weeks once they are no longer radioactive [12]. For such glasses, studies regarding dissolution mechanisms are required in order to better understand their in vitro and in vivo behavior. A recent study about dysprosium-containing lithium-borate glasses reported an amazing feature of these materials: even though the glass is degrading in the body, the radioisotope of rare earth element, reacting with the phosphate and other anions in the body fluids, forms an insoluble phosphate material which confines the radioisotope to the target organ [52]. It is possible that similar mechanisms occur with other glass compositions, too. Borate glass microspheres incorporating beta-emitting ^90^Y, ^153^Sm, ^165^Dy, ^166^Ho, and ^186^Re/^188^Re were tested on animals [12]; however, these glasses are still not available for commercial use [12].

Nogueira et al. [72] investigated a set of bioactive sol-gel glass containing radioisotopes of Zr, Ba, and Ho. The Zr and Ba allowed a better visualization of the seeds under radiographic imaging as these elements are good contrast agents. Ho was used because it emits higher energy than Sm, thus allowing to reduce the content of dopant in the bioactive glass structure and/or to develop a device that emits higher energy for the treatment of small tumors in shorter time [73]. Chemical and nuclear characterization analysis showed that ^166^Ho radionuclides were homogeneously distributed in the seeds [73]. The biodegradation process of glass was facilitated by neutron activation, which elicited deformation in the surface structure of the seeds [73]. Few years later, the same research group demonstrated that the Zr nuclide significantly increased the mass attenuation coefficient of Ho-Zr co-doped sol-gel glass seeds as compared to Zr-free materials. Thus, Ho-Zr-containing bioactive glass seeds offered a superior radiological response compared to that of Ho-doped glass seeds, thus increasing the radiological contrast [73].

In a very recent study, Piagentini Delpino and coworkers [74] developed new holmium-doped 58S-based glasses for bone cancer treatment by brachytherapy. The glasses, belonging to the system (100−x) (58SiO_2_-33CaO-9P_2_O_5_)-xHo_2_O_3_ (x = 1.25, 2.5, and 5 wt%) were characterized in terms of dissolution behavior, bioactivity, and cytotoxicity with pre-osteoblastic cells. Dissolution tests were performed in a Tris-HCl buffer solution according to ISO 10993-14: 2001. Moreover, the Arrhenius and Eyring equations were used to obtain some thermodynamic properties of glass dissolution. The bioactive behavior of the glasses was assessed by soaking tests in simulated body fluid (SBF), while cytotoxicity was determined by standard colorimetric 3-(4,5-dimethyl-2thiazolyl)- 2,5-diphenyl- 2H-tetrazolium bromide (MTT) assay. Interestingly, this study revealed a strong effect of the Ho content on the kinetics of the hydrolysis reaction, leading to a favored mechanism of dissolution with increasing dopant content. Despite this, most of the holmium ions remained embedded within the glassy matrix, thus preventing the development of cytotoxic effects due to high Ho concentration even in the composition with 5 wt.% of Ho_2_O_3_. No negative effects on the bioactivity mechanism were reported as all the systems were found to be as bioactive as the 58S sol-gel parent glass, used as a positive control system. In vitro cellular tests confirmed the cytocompatibility of all the materials analyzed, revealing an enhanced preosteoblastic cell proliferation as compared to the control (58S) [74].

Since the experimental procedures required for the preparation and characterization of radioactive glasses—especially in terms of biological efficacy—are quite complex, the use of molecular dynamics approaches for predictive and selective purposes have shown great promise in this field. A computational work developed by Sadeghi et al. [75] explained the interesting features of ^153^Sm in prostate brachytherapy once inserted within a biodegradable glass structure. Specifically, numerical simulations through Monte Carlo code were performed in order to analyze the relationship between dose and distance for a material doped with ^153^Sm. ^142^Pr beta emitter source was used as a benchmark to validate the simulation method accuracy and dose calculation. Additional data about other materials based on ^32^P and ^90^Sr/^90^Y beta emitters were also inserted for comparative purposes. It was possible to conclude that beta doses using ^153^Sm had a shorter effectiveness distance but the initial dose was higher compared to the other materials. These data also suggested that ^153^Sm would enable a less radiation effect on healthy tissues, reducing the side effects of radiotherapy and also decreasing the treatment time due to the higher initial dose [75].

**Figure 9:**
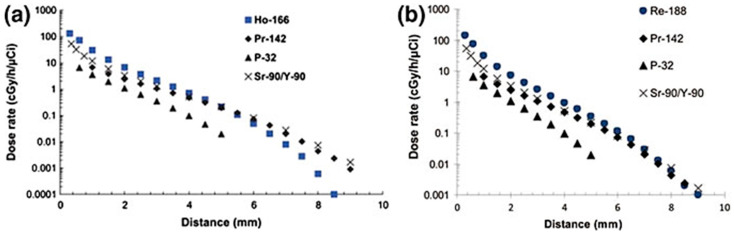
Comparison of the dose rate vs. distance for different glasses containing beta emitters. Figure adapted from Hosseini et al. [76] (**a**) and Khorshidi et al. [77] (**b**).

Hosseini et al. [76] and Khorshidi et al. [77] performed similar Monte Carlo code simulations to evaluate the beta dose of ^166^Ho-based and ^188^Re-based biodegradable glass seeds for hepatic cancer treatment. They concluded that both ^166^Ho and ^185^Re had a shorter dose distance as compared to ^153^Sm and a slightly higher initial dose, as shown in the Figure 9.

Despite these attractive properties, the use of rhenium radioisotopes carries some important drawbacks. ^186^Re and ^188^Re can emit both beta and gamma radiation during the radioactive decay, the latter inducing damage to healthy tissues and organs. Furthermore, the calculation of the radiation dose is more complex and should account for both beta and gamma emissions. Finally, the manufacturing of rhenium-containing glasses is more complex as compared to YAS glasses, requiring a single-step melting route.

In 2011, Christie et al. [24] studied, through molecular dynamics simulations, how yttrium contained in high-silica bioactive glass structures can influence the surface reactivity of the glass. Their purpose was to develop a highly bioactive glass which could release small amounts of yttrium in order to prevent the release of radionuclides into the bloodstream [24]. Their outcomes demonstrated that a low rate of yttrium leaching was related to high site-selectivity and clustering, which are believed to reduce the rate of yttrium transfer and release from the glass surface. At the same time, the limited network connectivity of the bioactive glass promotes the dissolution of the soluble species and enhances the glass network degradation [24]. For example, a satisfactory procedure may result from the incorporation of yttrium in some of the less bioactive compositions with high silica content. Y_2_O_3_ incorporation causes network fragmentation, which could offset the strong union between the silica fragments in the presence of yttrium, resulting in a glass composition with suitable bioactivity [24]. Furthermore, Christie et al. [78] also evaluated the effect of incorporating higher yttria amounts in the glass. The results illustrated the possibility of obtaining yttrium-containing glasses having enough biological activity to allow new tissue growth while being able to deliver higher radiation doses through a higher yttria content [78].

## 5. Conclusions and Outlook

Brachytherapy using biomedical radioactive glasses shows promise in combatting cancer but its use is still currently limited to the treatment of primary hepatocarcinoma and metastatic liver cancers. This therapeutic approach mainly aims at reducing the mass/size of tumor, in order to allow subsequent surgery, chemotherapy, or transplant, by using radioactive glass microparticles (size 20–30 μm) that are injected via the hepatic artery. As a result, these glass spheres accumulate in the tumor (embolization effect) and deliver a highly-localized dose of beta radiation to the targeted cancer tissue (radiation effect), thus killing the neoplastic cells.

To date, considering glass-matrix microspheres, TheraSphere^®^ microparticles, based on yttria-alumina-silica (YAS) glass, are the only clinically-used commercial product used for radioembolization. This YAS glass is activated by neutron irradiation to form ^90^Y radioisotope, with a short-range and short-half-life beta emitter, thus allowing safe delivery of a high dose of radiation to the tumor without major damages to surrounding healthy tissues.

Highly-durable glasses belonging to YAS compositions have been traditionally preferred for brachytherapy as compared to partially-soluble biomedical glasses, which may release radioisotopes in the body. However, a few works—mainly computational—have also been reported about the relationships among dose distance/intensity, potential therapeutic efficacy, and glass dissolution rate.

Combining radioactive therapy and bio-reactivity of glasses has a great potential to open new treatment perspectives for a broader range of cancer-associated diseases. In this regard, bioactive glasses are known to bond to bone via a surface hydroxyapatite layer, formed via an ion-exchange mechanism with body fluids, and to promote osteogenesis via the osteoinductive effects elicited by some ions released upon dissolution. Thus, considering diseases related to bone tissue, some special bioactive glass compositions could be developed to combine post-surgical radioactive treatment for killing residual cancer cells with bone regeneration in the defect, once the osseous tumor has been resected. New advancements in this field will be possible through a closer collaboration among glass chemists/technologists, clinicians, and nuclear scientists, further demonstrating how multi- and cross-disciplinary approaches are key to allow research to progress.

## Figures and Tables

**Table 1:** Characteristics of various beta-emitting radionuclides.

Radionuclide	Half-Life	Decay Energy/MeV	Decay Product
^90^Y	64.0 h	2.284	^90^Zr
^32^P	14.3 days	1.710	^32^S
^186^Re	90.6 h	1.076	^186^Os
^188^Re	17.0 h	2.119	^188^Os
^166^Ho	26.7 h	1.854	^166^Er
^153^Sm	46.7 h	0.817	^153^Eu
^45^Ca	162.7 days	0.26	^45^Sc
^40^K	1.25 × 10^9^ years	1.31	^40^Ca

**Table 2:** Application fields of microsphere technology in biomedicine and related commercial products, where available.

Material/Loaded Drugs/Biological Compounds	Clinical Application	Manufacturing Technology	Commercial Products	Ref.
Pure β-Tricalcium Phosphate(TCP) (≥99%)	Filler of oral and maxillofacial surgery bone defects	Sintering + crushing and sieving	Cerasorb^®^ (Curasan, Kleinostheim, Germany)	[34]
62.5% α-Tricalcium Phosphate (TCP), 26.8% dicalcium phosphate dihydrate, 8.9% calcium carbonate and 1.8% precipitated hydroxyapatite	Orthopedics: Bone graft substitute	Sintering + crushing and sieving	Calcibon (Biomet Deutschland GmbH, Berlin, Germany))	[35,36]
Poly(lactic-co-glycolic acid) (PLGA) loaded with Leuprolide acetate	Drug delivery: Endometrisis or Anemia prior to Uterine Fibroid surgery	Double emulsion-solvent evaporation method/self-healing encapsulation method	Lupron Depot (TAP Pharmaceutical Products Inc., Deerfield, IL, USA; Nihonbashi, Chuo, Tokyo, Japan)	[37,38]
Poly(lactic-co-glycolic acid) (PLGA) microspheres loaded with rhGH (recombinant human Growth Hormone)	Growth hormone regulator acting on skeletal and cell growth, protein, carbohydrate, lipid, mineral, and connective tissue metabolism	Spray freeze drying	Nutropin Depot^®^ (Genetech Inc., San Francisco, CA, USA; no longer available)	[39,40]
Poly(lactic-co-glycolic acid) (PLGA) loaded with Leuprorelin	Prostate cancer treatment, endometriosis treatment, breast cancer treatment, uterine fibroid treatment	Solvent evaporation encapsulation method	Enantone LP (Takeda Pharmaceutical Company Limited, Tokyo, Japan)	[41,42]
Poly(lactic-co-glycolic acid) (PLGA) loaded with Lanreotide	Neuroendocrine tumors, acromegaly treatment, carcinoid syndrome	Multiple-emulsion solvent evaporation method	Somatuline^®^ (IPSEN Pharma, Paris, France)	[43,44]
Poly(lactic-co-glycolic acid) (PLGA) loaded with Ocreotide	Acromegaly treatment, carcinoid syndrome, Neuroendocrine tumors, treatment of pituitary adenoma secreting TSH	O/W emulsion solvent evaporation technique	Sandostatin LAR (Novartis Farma, Origgio (VA), Italy)	[45,46]
70SiO_2_-30CaO glass (mol.%)	Dentistry/orthopedics	Sol-gel method	TheraGlass ^®^ (Imperial College, London, UK)	[47]
40Y_2_O_3_-20Al_2_O_3_-40SiO_2_ glass (wt.%)	Targeted HCC therapy (Intent or palliative treatment of liver cancer)	Flame spheroidization method	TheraSphere^®^ (Boston Scientific Corporation, Watertown, MA, USA)	[23,24,28,48]

## Data Availability

Data provided within the article or in the articles here reviewed.
